# Supplementary material for: The desmoglein 2 interactome in primary neonatal cardiomyocytes
Source: J Cell Sci. 2026 Feb 5;139(3):jcs264213. doi: 10.1242/jcs.264213 (PMC12912264; doi:10.1242/jcs.264213)
Supplement: Supplementary information [file joces-139-264213-s1.pdf]

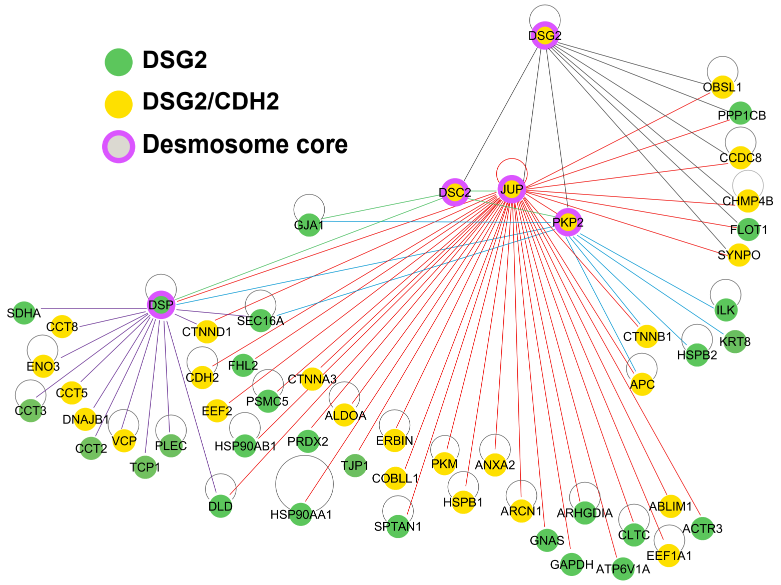

Fig. S1. DSG2 interactome hits that associate directly with desmo-somal proteins. Network assembled in IPA and organized manually around desmosome complex proteins. Published experimental data support all protein-protein interactions. Green circles signify hits unique to the DSG2 interactome; yellow circles mark hits shared with the CDH2 interactome. Desmosome proteins DSG2, DSC2, JUP, PKP2, and DSP are outlined in purple. Direct interactions with DSG2 are defined by gray lines; JUP, red lines; PKP2, blue lines; DSC2, green lines; DSP, purple lines.

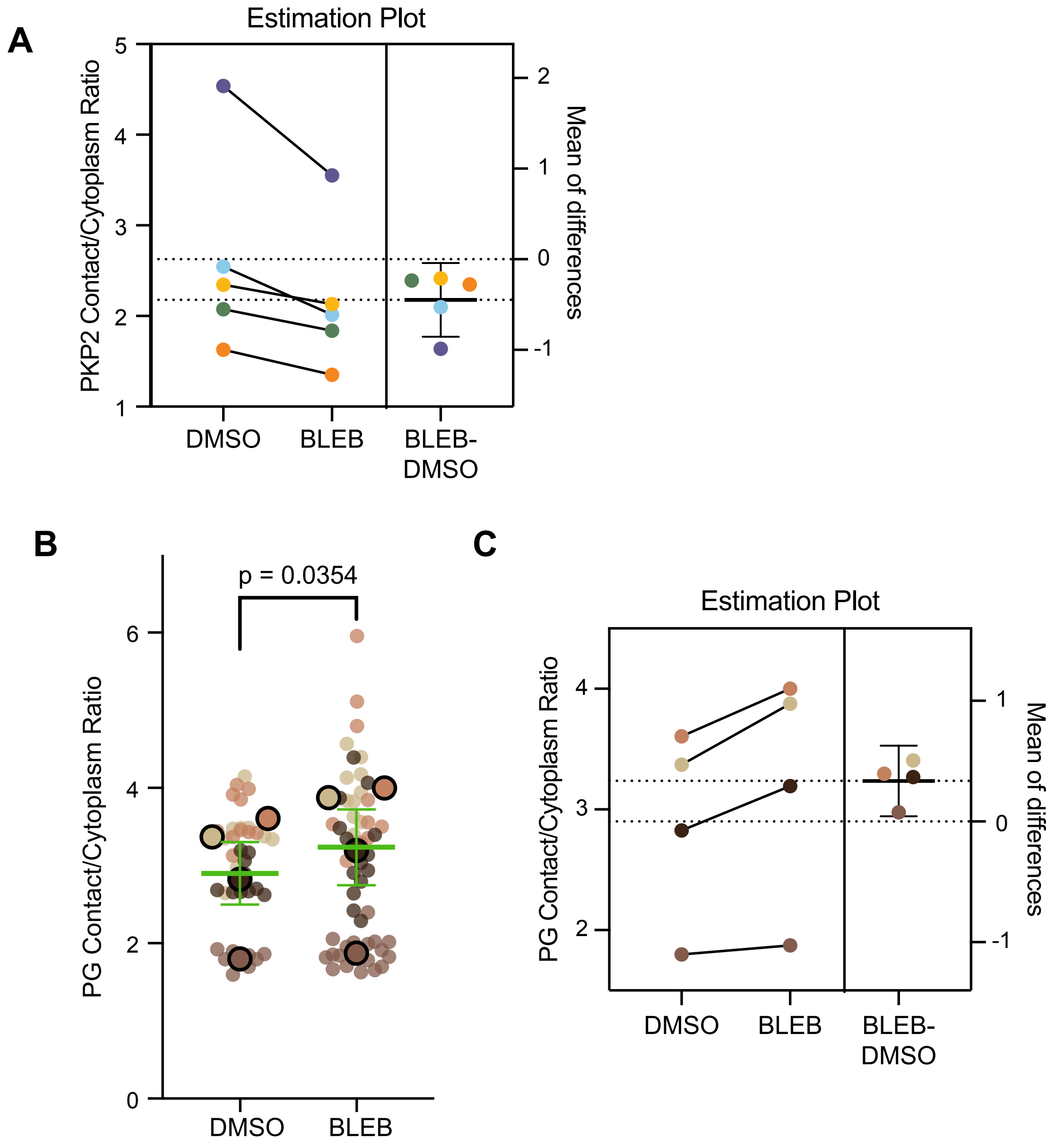

Fig. S2. Blebbistatin-dependent changes in PKP2 and JUP cell contact localization. (A) Paired t-test estimation plot for PKP contact/cytoplasm fluorescence ratio in cells treated with DMSO or blebbistatin (Fig. 8K). Left: The mean PKP contact/cytoplasm fluorescence ratio from each biological replicate is assigned a unique color and paired measurements (DMSO vs blebbistatin treatment) are connected by a black line. Right: The PKP contact/cytoplasm ratio difference between DMSO and blebbistatin treatments for each color-coded replicate are plotted. The mean and 95% confidence interval of the mean differences are shown as black bars. (B) JUP contact/cytoplasm ratio in cells treated with DMSO or blebbistatin. Data are presented as a SuperPlot (Lord et al., 2020), where individual measured JUP contact/cytoplasm fluorescence ratios (data points) from one biological replicate are plotted as circles, and each replicate is assigned a unique color (n = 4). The mean JUP contact/cytoplasm fluorescence ratio from each replicate is overlaid on the individual data points as a larger, color-matched circle outlined in black. The mean and standard error of the mean of the five replicates are shown as black bars. Blebbistatin treatment caused a significant increase in JUP enrichment at cell-cell contacts (p = 0.0354, paired t-test). (C) Paired t-test estimation plot for JUP contact/cytoplasm fluorescence ratio data in (B). Left: The mean JUP contact/cytoplasm fluorescence ratio from each biological replicate is assigned a unique color and paired measurements (DMSO vs blebbistatin treatment) are connected by a black line. Right: The JUP contact/cytoplasm ratio difference between DMSO and blebbistatin treatments for each color-coded replicate are plotted. The mean and 95% confidence interval of the mean differences are shown as black bars.

Journal of Cell Science • Supplementary information

Table S1.

Available for download at
https://journals.biologists.com/jcs/article-lookup/doi/10.1242/jcs.264213#supplementary-data

Table S2. List of unconnected DSG2-BioID hits – Fig. 5

ACOT2
C1orf198
C4of54
CAVIN4
CIAPIN1
CNN1
CYC1
EHBP1L1
FAM171A1
FAM171A2
FILIP1
GSTO2
HMGCS1
HTRA1
ITM2C
JCAD
JPH2
JPT1
LLPH
MLIP
MYOM1
MYZAP
PALM2-AKAP2
PAM
RAB11FIP5
RESF1
S100A11
SARS
SECISBP2
SERPINB6
SHB
SLITRK4
SMPX
SMYD1
SNRPC
SYNM
SYNPO2L
TMEM214
UBAP1

Journal of Cell Science • Supplementary information
